# Pre‐clinical and clinical evaluation of the HYPERSCINT plastic scintillation dosimetry research platform for in vivo dosimetry during radiotherapy

**DOI:** 10.1002/acm2.13551

**Published:** 2022-02-21

**Authors:** Imke Schoepper, Sonja Dieterich, Earl Alonzo Trestrail, Michael Sean Kent

**Affiliations:** ^1^ Department of Radiation Oncology University of California Davis School of Veterinary Medicine Davis California USA; ^2^ Department of Radiation Oncology University of California Davis Medical Center Sacramento California USA; ^3^ Pacific Crest Medical Physics Chico California USA

**Keywords:** In vivo dosimetry, plastic scintillators

## Abstract

**Purpose:**

The purpose of this work is to evaluate the Hyperscint‐RP100 scintillation dosimetry research platform (Hyperscint‐RP100, Medscint Inc., Quebec, QC, Canada) designed for clinical quality assurance (QA) for use in in vivo dosimetry measurements.

**Methods:**

The pre‐clinical evaluation of the scintillator was performed using a Varian TrueBeam linear accelerator. Dependency on field size, depth, dose, dose rate, and temperature were evaluated in a water tank and compared to calibration data from commissioning and annual QA. Angularity was evaluated with a 3D printed phantom. The clinical evaluation was first performed in two cadaver dogs, and then in three companion animal dogs receiving radiation therapy for nasal tumors. A treatment planning CT scan was performed for cadavers and clinical patients. Prior to treatment, the probe was inserted into the radiation field. Radiation was then delivered and measured with the scintillator. For cadavers, the treatment was repeated after making an intentional shift in patient position to simulate a treatment error.

**Results:**

In the preclinical measurements the dose differed from annual measurements as follows: field size −0.77 to 0.43%, depth dose −0.36 to 1.14%, dose −0.54 to 2.93%, dose rate 0.3 to 3.6%, and angularity −1.18 to 0.01%. Temperature dependency required a correction factor of 0.11%/°C. In the two cadavers, the dose differed by −1.17 to 0.91%. The device correctly detected the treatment error when the heads were intentionally laterally shifted. In three canine clinical patients treated in multiple fractions, the detected dose ranged from 98.33 to 103.15%.

**Conclusion:**

Results of this new device are promising although more work is necessary to fully validate it for clinical dosimetry.

## 1 INTRODUCTION

Radiation therapy and dosimetry are becoming increasingly complex due to advanced techniques including intensity‐modulated radiation therapy (IMRT) and volumetric modulated arc therapy (VMAT). Stereotactic high‐dose treatments further increase the need for accurate dosimetry to ensure patient safety. In vivo dosimetry has been shown to detect errors that could otherwise go unnoticed. The International Atomic Energy Agency (IAEA) therefore recommends in the Human Health Report #8 that in vivo dosimetry be routinely implemented as a clinical safety and quality measure. However, it is still not performed in many institutions due to often laborious or expensive techniques.^1–5^

Currently, there are few systems available for in vivo dosimetry: Thermoluminescent dosimeters (TLDs), GaFchromic film, diodes, Mosfets, optically stimulated luminescence dsimeters (OSLDs), and more recently, commercial EPID‐dosimetry systems. Plastic scintillators offer several characteristics which make them suitable for in vivo dosimetry: they are water‐equivalent, and independent of energy, dose, dose rate, and angularity.^6,7^ Unlike TLDs, film, Mosfet and diodes, scintillators are also capable of real‐time dosimetry. Further, they are very robust to radiation damage and can be easily cleaned between uses, allowing for use in multiple patients.

Because of their small size they are suitable for internal use, and they can be reused in multiple patients due to their long lifespan. Several studies found an inverse linear temperature dependency that required a correction factor.^8^, ^9^, ^10^, ^11^ One publication has addressed this problem and successfully introduced an approach to correct the dose instantaneously without applying a correction factor after measurement.^12^ A recent publication introduced a HYPERSCINT scintillation dosimetry research platform with a multi‐point setup (Medscint Inc., Quebec City, Canada) in comparison to a commercially available spectrometer. The spectrometer performed better in terms of spectral resolution, whereas the HYPERSCINT's detection efficiency was higher. Furthermore, the HYPERSCINT showed a better signal‐to‐noise ratio (SNR) and a better signal‐to‐background ratio (SBR). Measurements of dose, depth dose, full width at half‐maximum region of the beam profile and output factors were within 2.3% of the predicted dose.^13^ However, the published study used a modified system with the capability of multi‐point scintillation. Further, measurements were only performed in a preclinical setting.

The purpose of this study was to evaluate the single‐point HYPERSCINT scintillation dosimetry research platform (Medscint Inc., Quebec City, Canada) designed for clinical quality assurance (QA) for use in in vivo dosimetry. In a preclinical evaluation, the scintillator was characterized by taking measurements using a water tank and a 3D printed phantom. For the clinical evaluation, cadaver dogs as well as canine clinical patients with intranasal tumors were treated using IMRT or VMAT‐derived plans.

## 2 METHODS

All radiation exposures were delivered with a Varian TrueBeam^®^ linear accelerator (Varian Medical Systems, Palo Alto, CA), calibrated at 1 monitor units (MU) = 1cGy for a 10 cm × 10 cm field and 100 cm SSD at *d*_max_ using a water tank (RFA‐220, Scanditronix WellHöfer, Schwarzenbruck, Germany) with cylindrical ionization scanning chambers and certified therapy grade solid water (Gammex Model 457, Gammex Inc., WI) with PTW N23333 ionization chamber (CNMC, Nashville, TN). All measurements were performed using the single‐point BCF10 Hyperscint‐RP100 scintillation dosimetry research platform (Hyperscint‐RP100, Medscint inc., Quebec, QC, Canada).

The scintillator has a length of 3 mm and a diameter of 1 mm. The total length of the optical fiber connecting the detector to the camera was 20 m. Camera settings used for the measurements were set at a temperature of 10°C with an integration time of 1 s. Calibration of the probe was performed at room temperature according to the manufacturer's recommendations. Temperature of the room was measured using a commercially available thermometer (DBT‐100T digital barameter/Thermometer, CNMC, Nashville, TN).

Calibration was done according to the manufacturer's instructions. Briefly, a background measurement was performed. Then, a scintillation spectrum was acquired with the probe placed at the edge of a 4 cm × 4 cm electron field. Subsequently, a Cerenkov spectrum was acquired with the fiber positioned at *d*_max_, while the scintillator was outside the field and shielded with lead blocks. For the dose calibration, 500 MU were delivered at *d*_max_ of a 10 cm × 10 cm field using 6 MV photons. The software then calculated the calibration coefficients that were saved and used for subsequent measurements. This last step of the calibration was performed each day that the scintillator was used. The calibration was performed at room temperature and therefore varied slightly from day to day between 20.5°C and 21.2°C.

### 2.1 Preclinical evaluation

The dependency of the scintillator measurements on field size, depth, dose, dose rate, and temperature were evaluated by irradiating the scintillator in a commercially available water tank (RFA‐200, Scanditronix‐Wellhoefer, Bartlett, TN). The measured data were compared to calibration data from commissioning and annual QA testing which was done just prior to non‐clinical data collection.

The dependency on each factor was tested under standard conditions which were defined as follows: field size 10 cm × 10 cm at 100 cm SSD, depth *d*_max_, dose 100 MU, dose rate 600 MU/min, room temperature (= temperature used for calibration), photon energy of 6 MV, radiation angle 0°.

To evaluate the dependency of the dose measurement on the radiation beam angle a cylindrical 3D phantom with a diameter of 3 cm was printed using Acrylonitrile butadiene styrene (ABS) plastic to allow for dose buildup. The 3D phantom was centered at 100 source to axis distance (SAD) using orthogonal MV planar images. After inserting the scintillator, a dose of 100 MU was delivered from different angles. Standard conditions and all tested variables are shown in Table 1.

**TABLE 1:** Standard conditions and tested variables for each factor evaluated for the Hyperscint scintillation dosimetry platform

Factor	Standard conditions	Tested variables
Field size	10 × 10 cm	3 × 3 cm, 6 × 6 cm, 10 × 10 cm, 20 × 20 cm and 30 × 30 cm
Depth dose	1.5 cm	1.5 cm, 2 cm, 5 cm, 10 cm, 15 cm
Dose	100 MU	5 MU, 10 MU, 30 MU, 50 MU, 100 MU, 250 MU, 500 MU, 1000 MU
Dose rate	600 MU/min	100 MU/min, 200 MU/min, 300 MU/min, 600 MU/min
Temperature	Room temperature at which calibration had been performed	11.1°C, 15.8°C, 20°C, 25°C, 29.9°C, 35°C, 39.8°C
Energy	6 MV photons	6 MV photons
Radiation angle	0°	0°, 45°, 90°, 135°, 180°, 225°, 270°, and 315°

### 2.2 Clinical evaluation

This clinical portion of the study was approved by the Institutional Animal Care and Use Committee and Clinical Trials Review Board.

### 2.3 Cadaver heads and clinical patients

In order to implement the technique of in vivo dosimetry in animals, measurements were taken in two canine cadaver heads and three canine clinical patients with intranasal tumors.

The cadaver heads as well as the patients were placed in a previously described patient‐positioning mask device.^14^ A treatment planning computed tomography (CT) scan was performed using a diagnostic CT scanner (Lightspeed 16, General Electric, Waukesha, WI). IMRT or VMAT plans with a dose of 800 cGy per fraction were calculated in a commercially available treatment planning system using the Pencil Beam Anisotropic Analytical Algorithm (Eclipse Treatment planning system Version 15.5, Varian Medical Systems, Palo Alto, CA). The position of the scintillator was contoured on the treatment planning CT in the treatment planning system and the mean dose delivered to the scintillator structure was calculated.

Pre‐treatment QA was performed for each plan via a 2D diode array (MapCheck 3, Sun Nuclear, Melbourne, FL). Prior to treatment delivery, the intranasal temperature was measured with a standard digital patient thermometer (DT‐12 rapid digital thermometer, advanced Manufacturing Corporation, Taiwan) that is accurate within ± 0.2°C, and the scintillator was inserted into the planning target volume (PTV). A cone beam CT (CBCT) was performed to assure correct positioning and to document the exact position of the scintillator within the nasal cavity. The treatment plan was then delivered while measuring the dose with the scintillator. For clinical patients, measurements were taken on 3 consecutive treatment days. Cadaver heads were irradiated three times in a row after they had been positioned correctly via the CBCT. Subsequently, an intentional lateral shift of the cadaver head position was made to simulate a setup error in the range of daily uncertainties that moved the scintillator into the dose gradient region. The heads were again irradiated, and the dose measured three times in that shifted position.

To compare the measured dose to the expected dose, the CBCTs were matched with the treatment planning CT.

To compare the measured dose delivered to the intentionally shifted cadaver heads, the treatment in the shifted position was simulated in Eclipse and the dose recalculated.

Clinical patients had more intranasal mucous and soft tissue surrounding the scintillator which compromised the probe's visibility. Therefore, a radiopaque fiducial was attached (Figure 6a). Tests in the water tank confirmed that this fiducial did not influence the measurements.

### 2.4 Statistical analysis

All measurements in the water tank, the 3D phantom and the cadaver heads were performed in triplicate. A mean and a standard deviation were calculated, and the mean value was used to compare the measurements to the expected dose. To test for linearity of temperature dependency on dose, data were graphed and correlation statistics were done to check for goodness of fit using a commercially available software program (Microsoft Excel, Microsoft Corporation, Redmond, WA).

For clinical patients, the measurements were performed on three consecutive treatment days.

## 3 RESULTS

### 3.1 Preclinical evaluation

**FIGURE 1:**
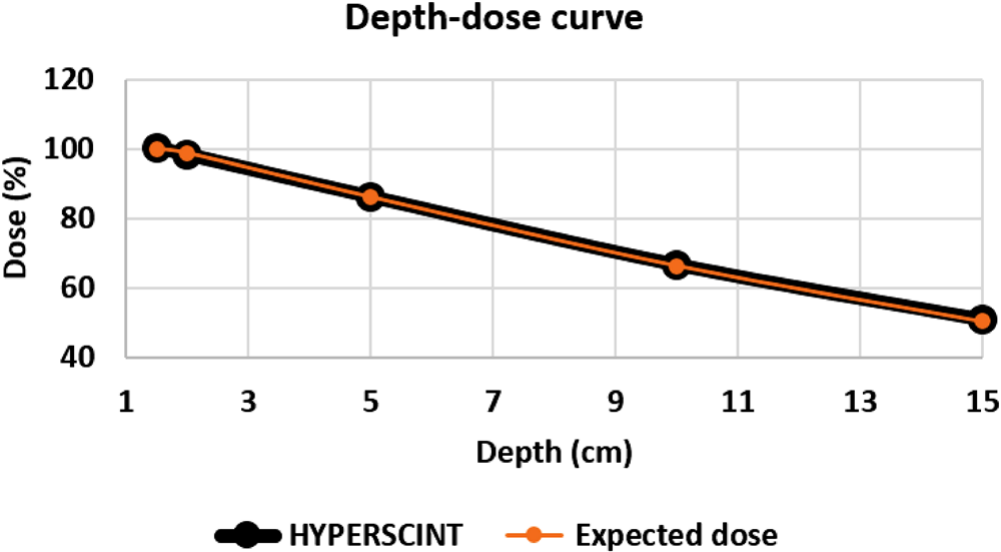
Depth–dose curve generated with the scintillator compared to the depth–dose curve from beam data. *Y*‐axis: dose measured in percent, *X*‐axis: depth of measurement in cm. 100 MU were delivered to different treatment depths at a dose rate of 600 MU/min using a 10 × 10 cm field with an SSD of 100 cm. Orange = expected dose, black = dose measured using the scintillator. Measurements were accurate with a dose difference ranging from 0.36% to 1.14%. The vertical error bars are not visible since the standard deviation of the measurements ranged from 0.03 to 0.14

**FIGURE 2:**
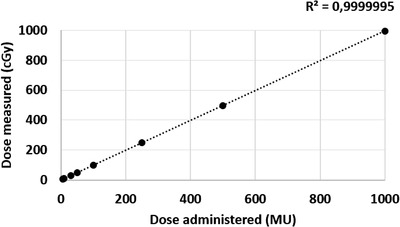
Dependency of dose measured on dose administered. *Y*‐axis: dose measured (cGy), *X*‐axis: dose‐administered (MU). Doses ranging from 5 MU to 1000 MU were delivered to *d*_max_ of a 10 × 10 cm field at a dose rate of 600 MU/min at room temperature. Vertical error bars (1 standard deviation) are not visible since the standard deviation ranged from 0.01 to 0.2. For doses of 10 MU or higher, the measured doses differed less than 1% from the expected dose. Only at a dose of 5 MU was the dose difference 2.93%

**FIGURE 3:**
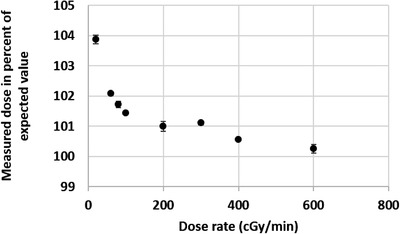
Dependency on dose rate. *Y*‐axis: measured dose (%) of expected dose, *X*‐axis: (MU/min) dose rate at which dose was delivered. Dose rate dependency of the Hyperscint scintillation dosimetry platform was tested using a water phantom by delivering 100 MU at different dose rates ranging from 20 to 600 MU/min using a 10 × 10 cm field at *d*_max_. At dose rates ≥ 100 MU/min, the detected doses ranged from 100.3% to 101.18% of the expected dose. At lower dose rates, the dose difference ranged from 1.45% to 3.6%. The vertical error bars show the standard deviation of the measurements that were taken in triplicate

**FIGURE 4:**
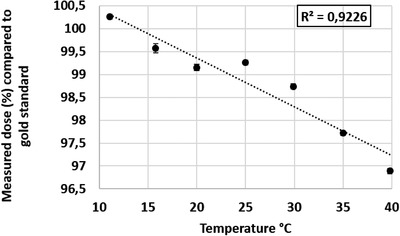
Dose dependency on the temperature. Dose dependency of the Hyperscint scintillation dosimetry platform was tested using a water phantom by delivering 100 cGy at *d*_max_ using a 10 × 10 cm field into water of different temperatures. The *Y*‐axis shows the measured dose as a percentage of the expected dose, and the *X*‐axis shows the water temperature (°C) that the measurement was taken at. An inverse linear relationship was detected and a correction factor of 0.11%/°C was calculated with a strong correlation. Vertical error bars show the standard deviation of the measurements. Horizontal error bars show the precision of the used thermometer of ±0.2°C

**FIGURE 5:**
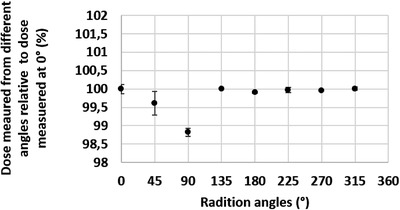
Dose dependency on radiation angle. *Y*‐axis: dose measured from radiation delivered at different angles relative to dose measured at 0°, X‐axis: angles from which radiation was delivered. Dose dependency on radiation angle was tested using a 3D printed phantom which was centered at 100 cm source to axis distance (SAD). Maximum dose difference from expected dose was −1.18%. The vertical error bars indicate the standard deviation of measurements which were taken in triplicate

For the tested field sizes between 3 cm × 3 cm and 30 cm × 30 cm, the dose difference measured with the scintillator ranged from −0.77% to 0.43%. For measurements taken at different depths between 1.5 cm and 15 cm, the dose difference ranged from 0.36% to 1.14%. Figure 1 shows the expected percent depth–dose curve in comparison with the percent depth–dose curve generated with the scintillator. Doses varying from 5 MU to 1000 MU were delivered. For doses from 10 MU to 1000 MU the detected dose difference was between −0.54% and +0.85%. At the lowest tested dose of 5 MU, the dose difference was 2.93% from that expected. Figure 2 shows the measured dose compared to the delivered dose. Dose was then delivered using dose rates between 20 MU/min and 600 MU/min. Table 2 and Figure 3 display the measurements for the different dose rates. At dose rates of 100 MU/min or higher, the delivered dose differences were 0.3%–1.18% from expected. At dose rates below 100 MU/min, the dose differences were higher showing a difference of 1.45%–3.6%. Dose dependency measurements showed an inverse linear relationship between dose and temperature of the water in the phantom between 11.1°C and 39.8°C. The higher the temperature, the lower the resulting dose measurements. A correction factor of 0.11%/°C was calculated (Figure 4) and showed an inverse linear relationship between temperature and measured dose and was highly correlated (*R* ^2^ = 0.92). The angular dependency of measured dose relative to the dose measured at 0° ranged between −1.18% and 0.01%. Figure 5 shows the results of the measurements from different angles.

**TABLE 2:** Measurements taken in triplicate under standard conditions for different dose rates

	Measured doses (cGy)					
Dose rate (MU/min)	1st	2nd	3rd	StDev (cGy)	Mean dose (cGy)	Relative dose (%)
20	103.74	103.84	104.02	0.14	103.87	103.6
60	102.04	102.04	102.15	0.06	102.08	101.81
80	101.81	101.72	101.61	0.1	101.71	101.45
100	101.40	101.48	101.44	0.04	101.44	101.18
200	101.10	101.08	100.82	0.16	101	100.74
300	101.16	101.09	101.06	0.05	101.1	100.84
400	100.56	100.56	100.57	0.01	100.56	100.3
600	100.42	100.19	100.17	0.14	100.26	100

### 3.2 Clinical evaluation

The intranasal temperature was taken in cadaver heads as well as clinical patients prior to irradiation. The Δ*T* (*T*_intranasal_ – *T*_calibration_) was calculated in order to apply the established correction factor.

### 3.3 Cadaver heads

6 MV VMAT plans using 1 arc were calculated and delivered to two cadaver heads. The prescribed dose was 800 cGy. For head 1, the measurements were between 844.89 cGy and 845.33 cGy with a mean dose of 845.13 cGy. For the second head, the measurements were between 837.55 cGy and 838.78 cGy with a mean dose of 838.01 cGy. The temperatures of the cadaver heads were 14.45°C and 15.75°C. This was colder than the probe's calibration temperature. After applying the temperature correction factor to the mean doses, the final measured dose differences were 0.73% for head 1 and −0.71% for head 2 compared to the calculated dose at the position of the probe.

After the cadaver heads had been intentionally shifted into an incorrect treatment position, the measured dose differed significantly from the prescribed dose in both cases. Thus, the treatment error was correctly detected. Both heads were shifted laterally, the first head was shifted 1 cm, the second head was shifted 2 cm. These shifts moved the scintillator out of the PTV and into the gradient area of the plan. The dose delivered at the shifted head position was simulated in the treatment planning software. The measured doses differed by 0.91% and −1.17% from the calculated dose. Table 3a shows the detailed measurements as well as the temperature correction for the cadaver heads treated in the correct position. Table 3b shows the results for the measurements performed in the shifted position.

**TABLE 3:** (a) Measurements taken in cadaver heads when treated in the correct position. (b) Measurements taken in cadaver heads after intentionally shifting the heads into a wrong position

(a)								
	Measured dose (cGy)	Mean dose (cGy)	Δ*T* (°C)	Correction factor (0.11×Δ*T*)	Absolute correction (cGy)	Corrected dose (cGy)	Expected dose (cGy)	Dose difference (%)
Head 1	845.25 845.33 845.06 844.89	845.13	−6.15	−0.68%	−5.8	839.33	833.27	0.73
Head 2	838.78 837.71 837.55	838.01	−4.85	−0.53%	−4.44	833.57	839.5	−0.71

(b)								
	Measured dose (cGy)	Mean dose (cGy)	Δ*T* (°C)	Correction factor (0.11×Δ*T*)	Absolute correction (cGy)	Corrected dose (cGy)	Expected dose (cGy)	Dose difference (%)
Head 1	372.19 371.63 371.10	371.64	−6.15	−0.68%	−2.53	369.11	365.77	0.91
Head 2	573.50 572.27 571.52	572.43	−4.85	−0.53%	−3.03	569.4	576.13	−1.17

Δ*T* (°C) = intranasal temperature − calibration temperature.

### 3.4 Clinical patients

Three small to medium‐sized client owned dogs with nasal tumors presenting for treatment to the UC Davis Veterinary Medical Teaching Hospital were enrolled in the study and treated with stereotactic radiotherapy to a dose of 2400 cGy delivered in three 800 cGy fractions. All dogs were anesthetized for the procedure. The anesthetic protocols consisted of a midazolam pre‐medication followed by a propofol induction and maintenance with isoflurane gas. Dog 2 also received Atropine. Dog #1 was a 29 kg poodle mix, dog #2 was a 33.5 kg Labrador retriever, and dog #3 was a 10.45 kg Boston terrier. In dog #2, the dose was delivered using a 2 arc VMAT plan, the other two dogs were treated with 11 field IMRT plans, which were delivered using a sliding window technique.

The measurements for each dog were taken on 3 consecutive treatment days. The scintillator was positioned into the PTV region which was confirmed with a CBCT. However, the scintillator's position within the PTV, and hence the expected dose, were different each treatment day.

**FIGURE 6:**
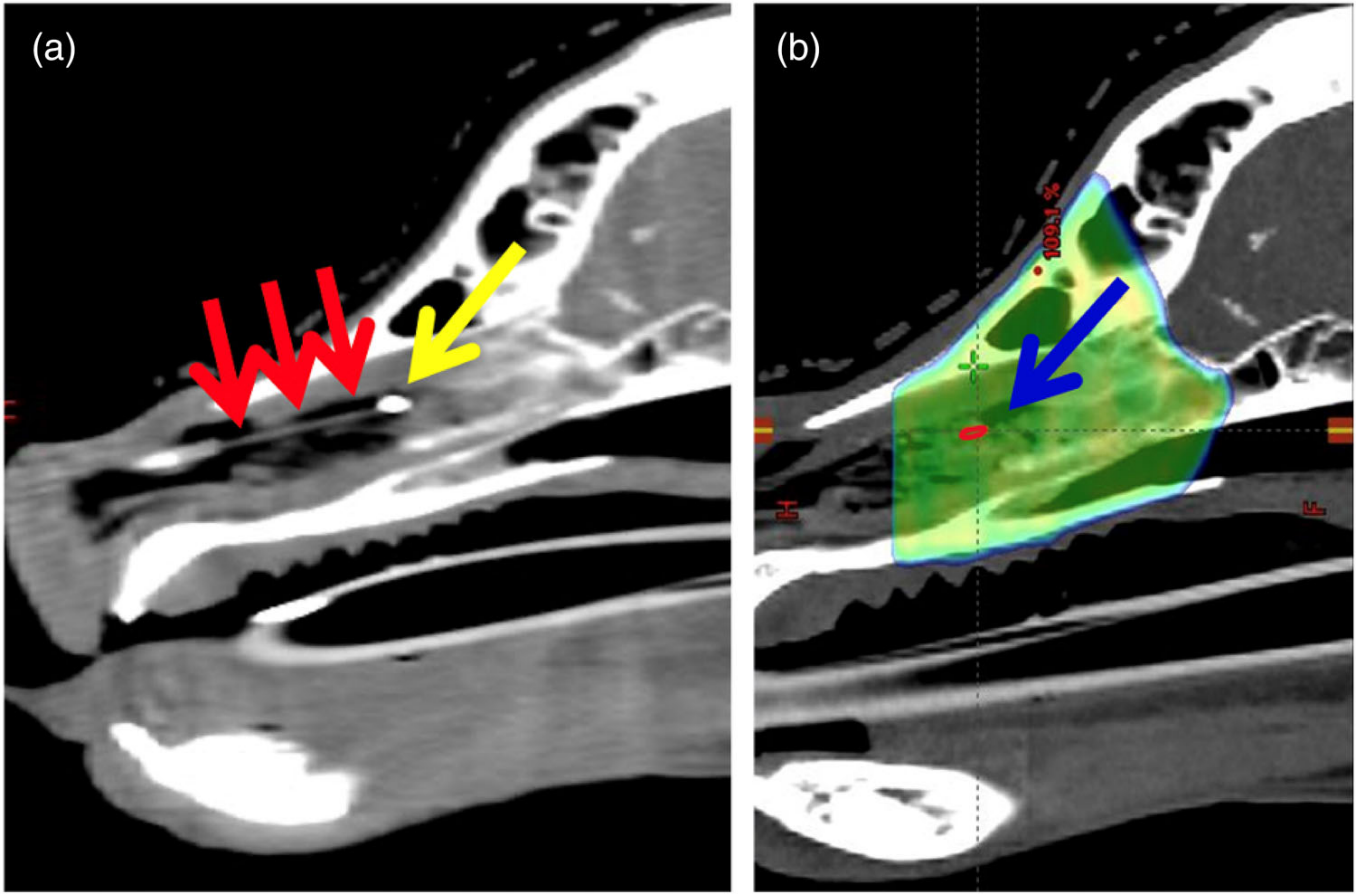
Cone beam computed tomography (CBCT) and treatment planning CT of a canine patient with an intranasal tumor. (a) CBCT showing the scintillator (red arrows) positioned within the nasal cavity. A fiducial (yellow arrow) was placed at the end of the scintillator. (b) Treatment planning computed tomography (CT) with the dose color wash of the treatment plan. The blue arrow indicates the position of the 3 mm long scintillator structure (red) that was contoured using the CBCT after matching the latter with the treatment planning CT. The mean dose delivered to the scintillator was then calculated by the treatment planning system

Figure 6a shows the sagittal view of a CBCT of dog #2 with the scintillator with the attached fiducial positioned within the nasal cavity. Figure 6b shows the treatment planning CT of the same dog with the dose color wash and the position of the scintillator structure drawn onto the CT scan.

In the clinical patients, the intranasal temperature was higher than the calibration temperature. The correction factor therefore increased the dose. Table 4 shows the detailed measurements and the temperature correction for the clinical patients. The corrected doses differed 3.15%, 0.37%, and −1.67% from the expected dose in dog 1. In the second dog, the measured doses were 2.55%, 1.12%, and 1.84% higher than the expected dose. In the third dog, the doses were 1.19%, 0.4%, and 1.75% higher than the expected dose. Overall, the dose difference in the three clinical patients was between −1.67% and 3.15%. None of the patients showed a clinically relevant increase in epistaxis after placing the scintillator in the nasal cavity.

**TABLE 4:** Measured dose, temperature correction, and final dose difference in three canine clinical patients

Dog	Day	Measured dose (cGy)	Δ*T* (°C)	Correction factor (0.11×Δ*T*)	Absolute correction (cGy)	Corrected dose (cGy)	Expected dose (cGy)	Dose difference (%)
Dog 1	1	842.3	17.2	1.89%	15.91	858.22	832	3.15
	2	831.09	17.03	1.87%	15.54	846.63	835.2	1.37
	3	814.15	16.7	1.84%	14.98	829.13	843.2	‐1.67
Dog 2	1	859.29	16.2	1.78%	15.29	874.58	852.8	2.55
	2	840.93	16.2	1.78%	14.97	855.9	846.4	1.12
	3	844.58	16.5	1.82%	15.37	859.95	844	1.89
Dog 3	1	823.44	15.9	1.75%	14.41	837.85	828	1.19
	2	817.9	16	1.76%	14.4	832.3	832	0.4
	3	840.42	15.4	1.69%	14.2	854.62	840	1.74

*Note*: *T* (°C) = intranasal temperature − calibration temperature.

## 4 DISCUSSION

In this study, the new Hyperscint scintillation dosimetry platform was evaluated in a preclinical and clinical setting. In the preclinical evaluation, the influence of field size, treatment depth, dose, dose rate, temperature, and treatment angle were evaluated. The small standard deviations of the measurements showed that the measurements were highly reproducible when performed in triplicate. For the tested field sizes, treatment depth, and radiation angle the dose differences were within ±1.2%.

At radiation doses between 10 cGy and 1000 cGy, the scintillator detected relative doses between 99.43% and 100.85% of the expected dose. Only at the lowest evaluated dose of 5 cGy, a larger dose difference of 2.93% was measured. This is likely due to the very short delivery time when the dose is administered at the standard dose rate of 600 MU/min. The 2.93% corresponds to an absolute dose difference of only 0.15 cGy which is considered clinically irrelevant.

Similarly, we observed that the scintillator performed more accurately at standard dose rates, but less accurately at extremely low dose rates. Figure 3 shows the measured dose plotted against the dose rate. The detected doses at dose rates above 100 MU/min were 0.3%–1.18% higher than the expected dose. When using dose rates below 100 MU/min, the dose difference was 1.45%–3.6%. Nevertheless, even in the most modulated VMAT plan, dose rates under 100 MU/min were used for only 0.2% of the total delivered dose. Therefore, this will not be clinically relevant in most cases treated with IMRT or VMAT techniques as shown by the accurate measurements in cadaver heads and clinical patients.

However, a low‐dose rate can also be generated by treating at an extended distance, for example, for total body irradiation. In such cases, the scintillator might perform less accurately, and further testing is needed.

As have other groups before, we detected an inverse linear temperature dependency of the system. The higher the Δ*T* (*T*_intranasal_ − *T*_calibration_) the lower the measured dose. A correction factor of 0.11%/°C was calculated. This is similar to correction factors reported by other publications which were between 0.09 and 0.75%/°C.^8^, ^10^, ^11^, ^12^ The Δ*T* in our clinical patients ranged from 15.4 to 17.2°C. Without the correction factor this could have potentially led to a dose measurement error between 1.7% and 1.9%.

The use of a temperature correction factor required measurements of the calibration temperature and intranasal temperature. In most intracavitary locations, the temperature can easily be measured. However, it would be more convenient to instantaneously correct for the temperature dependency without having to take these measurements. A previous publication introduced an approach that makes this feasible using the Hyperscint scintillation dosimetry research platform and the Hyperscint software.^12^ This feature could be used for future studies, especially if this tool were added to the standard manufacturer's software.

In the two cadaver heads, the measurements were again reproducible and accurate. The detected dose difference was between −1.17% and 0.91%. The shift of the heads into an incorrect treatment position was correctly identified by the scintillator.

For the clinical patients, all dose differences were between −1.67% and 3.15%.

There are few reports on plastic scintillators being used for intracavitary in vivo dosimetry in clinical patients. Wootton et al. used plastic scintillators to measure the rectal wall dose in five human patients treated for prostate cancer. The mean dose differences were −3.3% to 3.3%. The upper 95% confidence interval ranged from −0.6% to 8.7%, and the lower 95% confidence interval ranged from −1% to −5.9%.^15^

In comparison, the Hyperscint scintillation dosimetry platform performed better, but part of this might be due to the different treatment location. While the temperature is more consistent in the pelvic region, the setup accuracy is more challenging in this region than in the head. Furthermore, differences in the filling of the bladder and colon can account for small dose discrepancies as well. Noteworthy, Wootton et al. performed a total of 142 measurements, whereas we only report a total of 9 datasets.

There are other limitations to this study. We evaluated the system with only a single photon energy and therefore could not detect the scintillator's dependence on beam energy. The energy dependence as a function of field size was not assessed, which would be important for small fields. We also did not test the system at extended SSD distances. In this study, we only tested photons and not electrons. This system also used a single scintillator, which might be less likely to detect errors in a homogeneous dose volume. Further the tolerances of the temperature dependence of the probe could be further evaluated. Finally, a greater number of clinical patients with tumors in different anatomic locations would have also been useful and is planned for a future clinical evaluation of the system.

In conclusion, the Hyperscint scintillation dosimetry platform was easy to use and suitable for internal use. A temperature correction factor was necessary but could be avoided in the future by using a previously described approach. While larger studies are needed to confirm these results, this scintillator appears very promising for in vivo dosimetry and could therefore contribute to improved patient safety.

## AUTHOR CONTRIBUTION

Imke Schoepper: Substantial contributions to the conception of the work; acquisition, analysis, and interpretation of data. Drafting the work. Final approval of the version to be published.

## CONFLICT OF INTEREST

The authors declare that there is no conflict of interest that could be perceived as prejudicing the impartiality of the research reported.

## Data Availability

The data that support the findings of this study are available from the corresponding author upon reasonable request.
